# MARCKS as a Potential Therapeutic Target in Inflammatory Breast Cancer

**DOI:** 10.3390/cells11182926

**Published:** 2022-09-19

**Authors:** Maroua Manai, Ines ELBini-Dhouib, Pascal Finetti, Haifa Bichiou, Carolina Reduzzi, Dorra Aissaoui, Naziha Ben-Hamida, Emilie Agavnian, Najet Srairi-Abid, Marc Lopez, Fatma Amri, Lamia Guizani-Tabbane, Khaled Rahal, Karima Mrad, Mohamed Manai, Daniel Birnbaum, Emilie Mamessier, Massimo Cristofanilli, Hamouda Boussen, Maher Kharrat, Raoudha Doghri, François Bertucci

**Affiliations:** 1 Department of Medicine, Division of Hematology-Oncology, Weill Cornell Medicine, New York, NY 10021, USA; 2 Human Genetics Laboratory (LR99ES10), Faculty of Medicine of Tunis, University of Tunis El Manar, Tunis 2092, Tunisia; 3 Anatomic Pathology Department, Salah Azaiz Institute, Tunis 1006, Tunisia; 4 Biomolecules Laboratory of Venins and Theranostic Applications, Pasteur Institute of Tunis, Tunis 1002, Tunisia; 5 Predictive Oncology Laboratory, Centre de Recherche en Cancérologie de Marseille, Institut Paoli-Calmettes, Aix-Marseille University, «Equipe labellisée Ligue Contre le Cancer», 13009 Marseille, France; 6 Laboratory of Medical Parasitology, Biotechnology, and Biomolecules-LR16 IPT06, Institut Pasteur de Tunis, University of Tunis El Manar, Tunis 1002, Tunisia; 7 Department of Bio-Pathology, Paoli-Calmettes Institute, 13009 Marseille, France; 8 Laboratory of Neurophysiology Cellular Phytopathology and Biomolecules Valorisation (LR18ES03), Faculty of Sciences of Tunis, University of Tunis El Manar, Tunis 2092, Tunisia; 9 Department of Surgical Oncology, Salah Azaiez Institute, Bab Saadoun, Tunis 1006, Tunisia; 10 Mycology, Pathologies and Biomarkers Laboratory (LR16ES05), Faculty of Sciences of Tunis, University of Tunis El Manar, Tunis 2092, Tunisia; 11 Medical Oncology Service, Hospital of Ariana, Ariana 2080, Tunisia; 12 Medicine School, Aix-Marseille University, 13005 Marseille, France; 13 Department of Medical Oncology, Paoli-Calmettes Institute, 13009 Marseille, France

**Keywords:** inflammatory breast cancer, MARCKS, MPS treatment, mechanisms, PTEN, metastasis-free survival

## Abstract

Inflammatory breast cancer (IBC) is the most pro-metastatic form of breast cancer (BC). We previously demonstrated that protein overexpression of Myristoylated Alanine-Rich C Kinase Substrate (MARCKS) protein was associated with shorter survival in IBC patients. MARCKS has been associated with the PI3K/AKT pathway. MARCKS inhibitors are in development. Our objective was to investigate MARCKS, expressed preferentially in IBC that non-IBC (nIBC), as a novel potential therapeutic target for IBC. The biologic activity of MPS, a MARCKS peptide inhibitor, on cell proliferation, migration, invasion, and mammosphere formation was evaluated in IBC (SUM149 and SUM190) and nIBC (MDA-MB-231 and MCF7) cell lines, as well as its effects on protein expression in the PTEN/AKT and MAPK pathways. The prognostic relevance of MARCKS and phosphatase and tensin homolog (PTEN) protein expression as a surrogate marker of metastasis-free survival (MFS) was evaluated by immunohistochemistry (IHC) in a retrospective series of archival tumor samples derived from 180 IBC patients and 355 nIBC patients. In vitro MPS impaired cell proliferation, migration and invasion, and mammosphere formation in IBC cells. MARCKS inhibition upregulated PTEN and downregulated pAKT and pMAPK expression in IBC cells, but not in nIBC cells. By IHC, MARCKS expression and PTEN expression were negatively correlated in IBC samples and were associated with shorter MFS and longer MFS, respectively, in multivariate analysis. The combination of MARCKS-/PTEN+ protein status was associated with longer MFS in IBC patient only (*p* = 8.7 × 10^−3^), and mirrored the molecular profile (MARCKS-downregulated/PTEN-upregulated) of MPS-treated IBC cell lines. In conclusion, our results uncover a functional role of MARCKS implicated in IBC aggressiveness. Associated with the good-prognosis value of the MARCKS-/PTEN+ protein status that mirrors the molecular profile of MPS-treated IBC cell lines, our results suggest that MARCKS could be a potential therapeutic target in patients with MARCKS-positive IBC. Future preclinical studies using a larger panel of IBC cell lines, animal models and analysis of a larger series of clinical samples are warranted in order to validate our results.

## 1. Introduction

Inflammatory breast cancer is the most aggressive form of the disease with high metastatic risk [1] and accounts for 10% of BC deaths. The frequency of IBC is variable, ranging from less than 2% in Western countries to 5–10% in North Africa [2,3,4]. IBC is classified as T4d in the AJCC (American Joint Committee on Cancer, Chicago, USA) staging system. The positive diagnosis [2,5] is based on the rapid onset of breast erythema, edema and/or “peaud’orange”, occupying at least one-third of the breast, a duration of historyno more than six months, and the pathological confirmation of invasive carcinoma. Unfortunately, despite multimodality treatment, the survival rate of patients with IBC remains poor, with ~50% 3-year survival rate, much lower than the 85% rate among patients with stage III non-IBC (nIBC). To date, no IBC-specific targeted therapy has been approved by FDA, and the patients receive the same systemic treatment as patients with nIBC.

We previously generated the gene expression profiles of the largest clinical series of IBC samples compared to nIBC samples (137 IBC and 252 nIBC) [6], allowing to take into account in the supervised analysis the unbalance of molecular subtypes between IBC and nIBC. We identified a robust IBC/nIBC 79-gene expression signature independent from the molecular subtypes in which *MARCKS* was the second gene most overexpressed in IBC vs. nIBC. In another study, we evaluated, using immunohistochemistry, MARCKS protein expression in 133 IBC and 369 nIBC clinical samples. MARCKS protein expression was associated with IBC phenotype independently from molecular subtypes and other clinicopathological variables. Interestingly, in IBC only, MARCKS expression was associated with poor metastasis-free survival (MFS), suggesting that MARCKS might be a therapeutic target in IBC [7]. 

MARCKS is a substrate of protein kinase C (PKC), and its activation induces its detachment from the membrane and binding to a single actin filament. These conditions lead to the tridimensional rearrangement of the cytoskeleton, inducing cell motility, cell division, malignant transformation, and aggressive signal transduction pathways activation [8,9,10]. In some tissues, MARCKS phosphorylation is regulated by other kinases, such as Rho kinases and mitogen-activated protein kinases(MAPKs) [11,12], whose activation has been linked to the metastatic process and found to be overexpressed in IBC [7]. In the literature, inhibition of MARCKS using MPS (MARCKS phosphorylation site domain) peptide showed promising results in different cancers with inhibition of tumor growth and metastases in vivo [13,14], but no study was reported in breast cancer. 

The objectives of our present study were: (i) to determine in vitro the eventual inhibitory effects of MPS on the cell proliferation, migration and invasion, and mammosphere formation in IBC vs. nIBC cells, (ii) to investigate in vitro the distinct molecular mechanisms of action of MPS in IBC compared to nIBC cells, and (iii) to determine the clinical relevance of the target protein component(s), MARCKS and PTEN, in IBC vs. nIBC patients by analyzing diagnostic tumor samples. To our knowledge, our study is the first to target MARCKS in IBC and to investigate its functional role in the disease aggressiveness comparatively in IBC and nIBC. Our results suggest that MARCKS is a potentially druggable target in IBC.

## 2. Materials and Methods

### 2.1. Cell Culture and MARCKS Inhibitor Treatment

Four BC cell lines were tested: two IBC cell lines (SUM149 triple negative (TN), and SUM190 non-TN) and two nIBC cell lines (MDA-MB-231 TN, and MCF7 non-TN). SUM149 triple negative (TN) IBC cells were cultured in Ham’s F12 medium with 10% FBS, 1% penicillin/streptomycin, 1% L-glutamine, 100 μg/L hydrocortisone, and 5 μg/mL insulin. The MDA-MB-231TN nIBC cells were grown in a DMEM-F12 medium with 10% FBS, and 1% penicillin/streptomycin. Cells were periodically verified for mycoplasma. The SUM cell lines were kindly provided by Dr. Ethier (https://sumlineknowledgebase.com/, accessed on 25 January 2000) and MDA-MB-231 and MCF7 were obtained from ATCC (LGC Standards, Molsheim, France). IBC and nIBC cells were grown at 37 °C in a humidified atmosphere of 5% CO_2_. MPS and MPS mutated (MPSm, used as negative therapeutic control), were synthesized at Covalab, Bron, France (detailed sequences are available as supplementary material). Identity of cell lines was verified every 6 months using genotyping.

### 2.2. Cell Viability, Proliferation, and Colony Formation Assays

In brief, we first measured the half-maximal inhibitory concentration (IC50) of each tested peptide, MPS and MPSm by MTT assay (3-(4,5-dimethylthiazol-2-yl)-2,5-diphenyltetrazolium bromide). A total of 0.8 × 10^3^ to 3 × 10^3^ cells were added into a 96-well plate and left overnight, and cells were treated with MPS and MPSm for three days with doses ranging from 0 to 50 µM (3 replicates). Next, we added 20 μL MTT solution per well, plates were incubated for 3 to 4 h at 37 °C, the medium was removed, 150 µL of DMSO was added to each well and absorbance was measured using the plate reader at 490 nm. For the evaluation of IC50, we used GraphPad Prism software (version 9.4.0, Chicago, IL, USA). For colony formation assay, IBC and nIBC cells were seeded to 6-well tissue culture dishes (0.5 × 10^3^ to 1 × 10^3^ cells) and left overnight, then treated with the MPS and MPSm peptides with IC50 (25 µM) and incubated for 7 to 14 days. Colonies were washed gently with PBS, fixed with methanol for 30 min at 4 °C and finally stained with crystal violet: methanol at the ratio of 1:1. We counted the number of colonies using ImageJ (1.8.0_172, LOCI, University of Wisconsin) and analyzed using GraphPad Prism software.

### 2.3. Scratch Wound-Healing Assay 

IBC and nIBC cells were seeded into 6-well tissue culture dishes and grown to confluence in triplicate. Once the monolayer wells became confluent, we wounded linearly using a pipette tip and washed three times with PBS. After the addition of MPS and MPSm (25 µM) as a single treatment, cell migration was observed and photographed at regular intervals at 0 h, 12 h, 24 h, and 48 h after the scratch. The measurement of number of cells migrated into the cell-free zone was carried out under a light microscope at 12 h, 24 h, and 48 h compared to 0 h. 

### 2.4. Transwell Migration and Invasion Assay

We performed a second cell migration assay in triplicate using a chemotaxis chamber (24-well inserts, 8-µm pore size). After treatment with MPS and MPSm (25 µM), cells were harvested and resuspended in serum-free media containing 1 mg/mL of bovine serum albumin. 1 × 10^5^ cells/350 μLwere plated in the upper chambers, and bottom wells were filled with 750 μL of media that included 2% FBS, and 100 ng/mL EGF as an attractant to induce migration or invasion. Eight to ten hours later, at 37 °C for migration, membranes were fixed and stained with Dip-Quik solutions (Thermo Scientific, Waltham, MA, USA), then scanned for evaluation by the ImageJ program and analyzed using GraphPad Prism software. For invasion assay, a Matrigel (Corning, NY, USA) invasion chamber was used.

### 2.5. Mammosphere Formation Assay

Cells were treated with MPS and MPSm for 24 h in 6-well monolayer plates, trypsinized, and seeded into single-cell suspensions in 6-well culture plates with ultra-low attachment to prevent cell adhesion at a density of 1000 cells/mL in serum-free MammoCult^TM^ supplemented with 1% L-glutamine, 1% penicillin/streptomycin, 2% B27, 20 ng/mL EGF and 20 ng/mL FGFb. After 7 days of culture with the presence of MPS and MPSm, mammospheres were treated with MTT (1 mg/mL) and incubated at 37 °C for 2 h. Next, plates were scanned, and mammospheres were counted using ImageJ and analyzed using GraphPad Prism software.

### 2.6. Western Blot Analysis

Cultured and treated cells after 24 h were washed twice with PBS before lysing with M-PER Mammalian protein extraction reagent mixed with HALT^TM^ protease inhibitor cocktail (1:100 dilution). Lysates were then collected, then vortexed every 3 min for 20 min by keeping the samples on ice while the vortex steps and centrifuged for 15 min at 4 °C and 15,000 rpm to finally collect the supernatant. For Western blot, we used 10 to 20 μg of protein that were denatured at 70 °C during 10 min, then migrated in SDS-PAGE and transferred in Polyvinylidene difluoride (PVDF) membrane. We evaluated the effect of MPS and MPSm on expression of MARCKS and phosphorylated-MARCKS (p-MARCKS), phosphatase and tensin homolog (PTEN), AKT and phosphorylated-AKT (p-AKT), phosphorylated- mitogen-activated protein kinase (p-MAPK), cleaved caspase 3, and cleaved-Poly-ADP-ribose polymerase (cleaved-PARP) for apoptosis. We used β-actin to demonstrate equal loading and to normalize data. The phosphorylated forms were normalized to the respective total protein when available (MARCKS and AKT). ImageJ analysis software was used to evaluate the amounts of the target proteins normalized according to control. The results were obtained from triplicate independent experiments, and the comparison between the different treatment conditions was carried out using the *t*-test.

### 2.7. Patients and Samples

We collected pre-therapeutic diagnostic formaldehyde-fixed, paraffin-embedded (FFPE) tumor samples (180 IBC and 355 nIBC) from patients treated at Salah Azaiez Institute (SAI) of Tunis (Tunisia), and Paoli-Calmettes Institute (IPC) of Marseille (France). The clinicopathological data were retrospectively collected from our institutional medical registries and pathology databases. The inclusion criteria included pathologically confirmed IBC (AJCC T4d), availability of pre-therapeutic diagnostic FFPE tumor samples, clinicopathological annotations including treatment and follow-up, and patient’s written informed consent. The patients had received standard multidisciplinary treatment in both hospitals, including neoadjuvant and/or adjuvant chemotherapy in all cases, hormone therapy and anti-HER2 treatment when indicated, and surgery and adjuvant radiotherapy. This study was approved by the Institutional Review Boards of the two participating centers (n°2015-1618 for SAI and n°15-001 for IPC).

### 2.8. Immunohistochemistry (IHC) Analysis

The TMA construction of IBC and nIBC clinical samples was carried out as previously reported [15]. MARCKS protein expression was analyzed as previously reported [7] in the whole series with a positivity cut-off of 1% and more of positively stained tumor cells. PTEN protein expression was analyzed on TMA slides for 54 IBC and the 231 nIBC using standard IHC protocols. IHC was performed on 4-μm sections. Paraffin sections were pretreated in PT Link pH9. PTEN staining was carried out with the rabbit monoclonal antibody, anti-PTEN that was diluted at 1:100. A cut-off of 100 Quick Score (QS) was used for positive PTEN staining (QS = P × I, ranging between 0–300). The stainings were independently analyzed by two experienced breast pathologists (RD and KM) using light microscopy. 

### 2.9. Statistical Analysis

Cell proliferation, colony formation, migration, invasion and mammosphere formation rates were summarized with descriptive statistics and box plots for each treatment group. A two-tailed unpaired Student *t*-test was used for statistical analysis using GraphPad Prism software (version 9.4.0, Chicago, IL, USA). Regarding the analysis of clinicopathological variables, data were summarized by numbers and percentages for categorical variables and median and range for continuous variables. Mann-Whitney and Fisher’s exact tests, when appropriate, were used to analyze correlations between patients’ groups and clinicopathological variables. Uni- and multivariate analyses regarding the IBC/nIBC distinction were carried out using logistic regression analysis using the glm function and the significance was estimated by specifying a binomial family for model with a logit link. MFS was calculated from the date of diagnosis until the date of distant relapse or death from any cause, whichever occurred first. Follow-up was measured from the date of diagnosis to the date of last news for event-free patients. Survivals were calculated using the Kaplan-Meier method and curves were compared with the log-rank test. Uni- and multivariate prognostic analyses for MFS sere carried out using the Wald test; the variables tested included the patients’ age (continuous value), the pathological type (ductal, lobular, mixed, other), the pathological grade (1, 2, 3), the molecular subtype (HR+/HER2−, HER2+, TN), and for nIBC the pathological tumor size (PT1, pT2, pT3) and axillary lymph node status (negative, positive). All variables with a *p*-value ≤ 0.05 were included in the multivariate analysis. The correlation between MARCKS expression as discrete value (cut-off 1%) and PTEN expression was measured using the Mann-Whitney test when considering PTEN as continuous values (Quick Score) and using the logistic regression (logit link test) when considering PTEN as discrete values (cut-off Quick Score 100%). A *p*-value ≤ 0.05 was considered as significant and was represented in the figures with (*) for *p* ≤ 0.05, (**) for *p* ≤ 0.01, (***) for *p* ≤ 0.001, and (****) for *p* ≤ 0.0001. Analyses were performed by the survival package (version 2.43-3) in the R software (version 3.5.2; www.cran.r-project.org/, accessed on 7 January 2019; Auckland, New Zealand; www.cran.r-project.org/, accessed on 7 January 2019).

## 3. Results

### 3.1. MPS Inhibits Proliferation of IBC Cells at Lower Doses Compared to nIBC

**Figure 1:**
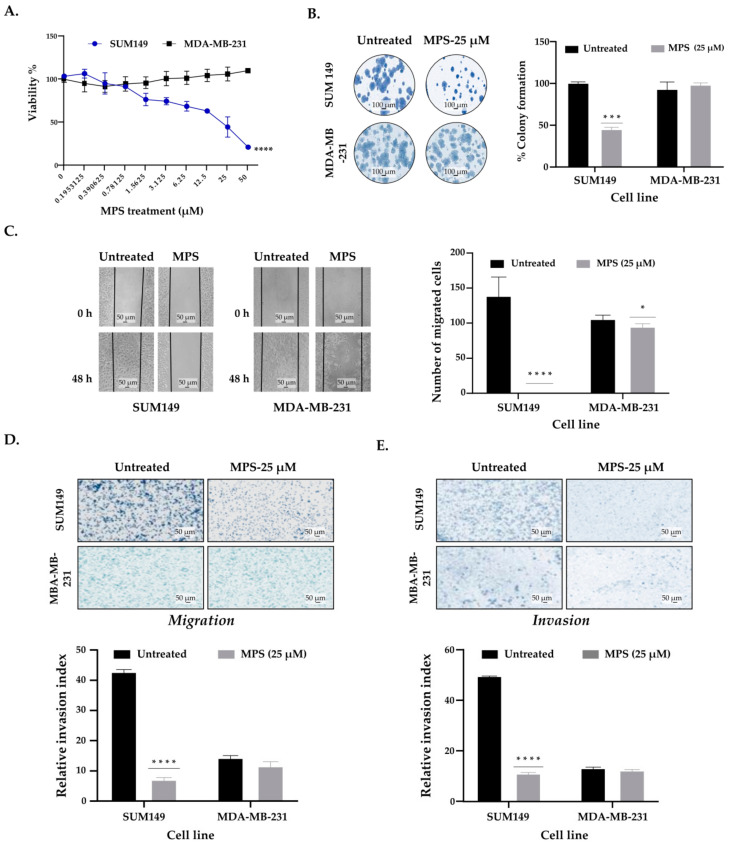
MARCKS promotes cell proliferation and motility of IBC. (**A**) MTT assay showed that MARCKS inhibition using 25 µM MPS peptide induced cell growth inhibition only in SUM149 compared to MDA-MB-231 cells. (**B**) MPS reduced colony formation of SUM149 cells, but not in MDA-MB-231: representative images (left) and box plots (right). (**C**) Representative images of migration in scratch/wound-healing assay to evaluate the inhibitory effect of MPS peptide on IBC vs. nIBC cell migration (left). Each confluent monolayer was wounded linearly then treated with MPS (25 µM), cell morphology and migration were observed and photographed at regular intervals, and the number of the migrated cells into the cell-free zone was calculated at 48 h compared to 0 h. (**D**) Similar to (**C**) but using the transwell migration assay. Migration was stained and measured after 24 h: representative images (top) and box plots (bottom). (**E**) Representative images of Matrigel invasion in chambers demonstrating the inhibitory effect of MPS in IBC cells (top) and box plots (bottom). Data were represented as mean ± SD. * for *p* ≤ 0.05, *** *p* ≤ 0.001 and **** *p* ≤ 0.0001, (3 replicates).

We first tested in vitro the effect of MARCKS inhibition on cell proliferation by treating IBC (SUM149) and nIBC (MDA-MB-231) cells for 24 h with MPS and MPSm. MARCKS treatment with 25 µM inhibited the proliferation of IBC (SUM149) significantly more when compared to nIBC cells (MDA-MB-231; *p* < 0.0001; Figure 1A). The IC50 was 25 µM for SUM149 cells and superior to 50 μM for MDA-MB-231). Additionally, we performed a colony formation assay: MPS treatment induced a dramatic decrease of the number of colonies in SUM149 cells (treated vs. untreated: *p* = 0.0004; Figure 1B), without any modification in MDA-MB-231 cells (treated vs. untreated: *p* = 0.376). Of note, the number of colonies was not different between the untreated IBC vs. nIBC cells (*p* = 0.218). Concerning the MPSm treatment, we did not find any significant effect on proliferation and colony formation in both IBC and nIBC cells (Supplementary Figure S1A,B). Similar results were observed with two other BC cell lines: decrease of cell proliferation and colony formation in the SUM190 IBC cell line after treatment with MPS, but no effect on the MCF7 nIBC cell line (Figure S2A,B).

### 3.2. MPS Inhibits the Invasiveness and Cell Motility in IBC Cells 

We determined in vitro and in silico whether MARCKS was involved in cell invasiveness and motility in IBC and nIBC cells. 

First, we used the scratch wound-healing and transwell assays to evaluate the migration and cell motility. In both migration assays (Figure 1C,D), we found a dramatic decrease in cell migration after MPS treatment in SUM149 cells (treated vs. untreated: *p* = 0.002 for the scratch wound-healing assay and *p* < 0.0001 for the transwell assay). By contrast, in MDA-MB-231 cells, MPS treatment decreased slightly the cell migration in the scratch wound-healing assay only (*p* = 0.049) and did not modify it in the transwell migration assay (*p* = 0.132). Of note, and as expected, there was a significantly larger cell migration in SUM149 compared to MDA-MB-231 cells in untreated conditions (*p* < 0.0001 for both assays). 

Next, to assess the effect of MPS on cell invasiveness, we performed the transwell invasion assay in a chamber using Matrigel. We found a significant inhibition of cell invasion by MPS in IBC cells (*p* < 0.0001; Figure 1E) but no effect on nIBC cell invasiveness (*p* = 0.94). As expected, we found higher invasiveness in IBC cells than in nIBC cells in untreated conditions (*p* < 0.0001). MPSm treatment did not affect cell migration and invasion in both IBC and nIBC cells (Figure S1C,D). We also validated these observations using SUM190 and MCF7 cell lines (Figure S2C,D). Altogether, these results suggested that MARCKS is a mediator of invasion and migration in IBC cells. In order to expand these experiments, we analyzed the MARCKS protein expression in invasive margins of 19 additional MARCKS-positive IBC samples profiled in a previous publication [7] using informative standard slides (where the sections presented invasive margins). Interestingly, we found an overexpression of MARCKS in cancer cells located in invasive margins in 18 of 19 samples (95%). Furthermore, some samples displayed an overexpression within tumor emboli (Figure S3). We also searched for an eventual co-expression of epithelial-mesenchymal transition (EMT) markers with *MARCKS* expression in our institutional transcriptomics data of 71 IBC clinical samples [6]. As shown in Table S1, using logistic regression (logit link test) stratified upon the molecular subtypes, we found strong positive correlations between MARCKS expression and expression of genes involved in EMT (*TWIST1, TWIST2, ZEB1, ZEB2, VIM*) and between *MARCKS* expression and a gene expression signature related to EMT [16].

### 3.3. MPS Impairs the Mammosphere Formation in IBC Cells 

**Figure 2:**
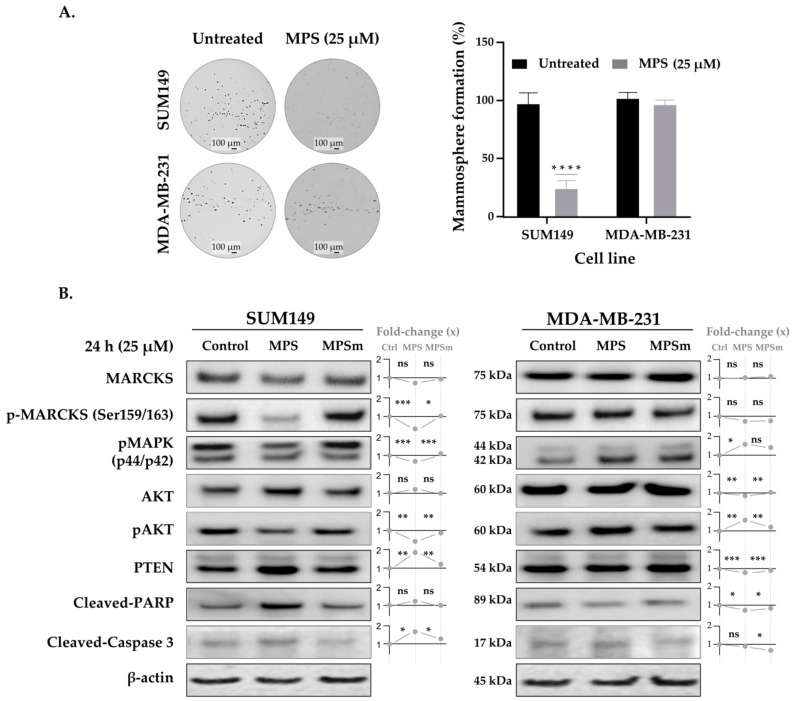
MARCKS promotes mammosphere formation of IBC cells compared to nIBC and Western blot analysis. (**A**) Representative images (left) showing that MARCKS inhibition using MPS peptide impaired mammosphere formation in SUM149 compared to MDA-MB-231 cells (magnification ×10); box plots are shown (right). (**B**) Western blot analysis. SUM149 and MDA-MB-231 cells were treated with 25 µM MPS and MPSm, and after 24 h of incubation, the protein was extracted, and the expression was analyzed using Western blot. The results were quantified and normalized using the protein expression level of β-actin. The phosphorylated forms were normalized to the respective total protein when available (MARCKS and AKT). To the right of each blot, the fold-change of each treatment (MPS and MPSm) is relative to the untreated condition (control). The adjusted *p*-value using Bonferroni (indicated with “ns” or stars) is for the *t*-test comparing expression in between MPS and control conditions and between MPS and MPSmconditions. In SUM149, we found a decrease of pMARCKS protein expression in MPS-treated cells compared to untreated and MPSm-treated cells, inducing then a decrease of pAKT and an increase of PTEN expressions and an increase of apoptotic markers expression (cleaved-PARP and cleaved-Cas3), and a decrease of pMAPK expression explaining in part the inhibition of primary mammosphere formation in IBC cells. By contrast, no similar change in expression was observed in MD-MB-231 cells. Data were represented as mean ± SD. **** *p* ≤ 0.0001, *** *p* ≤ 0.001, ** *p* ≤ 0.01, * *p* ≤ 0.05, ns: *p* > 0.05, (3 replicates).

IBC is known to be more associated than nIBC with tumor cell stemness and the formation of mammospheres [17,18]. To further investigate the role of MARCKS in the high metastatic propensity of IBC, we evaluated the effect of MPS in inhibiting primary mammosphere formation in both IBC and nIBC cells. The cell lines were treated in vitro with 25 µM of MPS and MPSm for seven days, and the formed spheres were counted. MPS significantly impaired mammosphere formation in SUM149 (treated vs. untreated, *p* < 0.0001; Figure 2A). By contrast, no significant difference was found between treated and untreated nIBC cells (*p* = 0.309), nor between untreated IBC and nIBC cells (*p* = 0.716). By contrast, no significant effect of MPSm treatment was found in both SUM149 and MDA-MB-231 cells (Figure S1E). As shown in Figure S2E, similar results were observed with SUM190 and MCF7 cell lines. In order to expand these experiments, we searched for an eventual co-expression of stemness markers with MARCKS expression in our transcriptomics data of 71 IBC clinical samples [6]. Logistic regression stratified upon the molecular subtypes identified strong positive correlations between MARCKS expression and expression of the *ALDH1A1* gene, which encodes the aldehyde dehydrogenase 1 (ALDH1) enzyme, a marker of normal and malignant human mammary stem cells [19], and between MARCKS expression and a Lim’s differentiation score closer to mammary stem cells than mature luminal cells [20]. By contrast, no correlation was found between *CDH1* expression and the CD44+/CD24– profile (Table S1).

### 3.4. MPS Downregulates MAPK in IBC 

Using Western blot analysis, we investigated the mechanisms of action of MPS in IBC compared to nIBC cells, using the cell protein lysates after a 24 h treatment with 25 µM MPS and MPSm. The results were quantified and normalized using the protein expression level of β-actin. As shown in Figure 2B, MPS had an inhibitory effect on p-MARCKS expression in SUM149 cells compared to MDA-MB-231 in which MPS had no effect on MARCKS and p-MARCKS proteins expression. MAPK is considered an upstream regulator of MARCKS, inducing its phosphorylation [21]. Our Western blot analysis showed that, unexpectedly, both p42 MAPK (MAPK1/MK01) and p44 MAPK (MAPK3/MK03) were significantly decreased in SUM149 after MARCKS inhibition, whereas an opposite effect was shown in MDA-MB-231 (Figure 2B). Furthermore, untreated SUM149 cells presented a higher level of pMAPK activity as compared to untreated MDA-MB-231 cells (Figure 2B). Similar observations were carried out using SUM190 and MCF7 cell lines (Figure S2F), with an MPS-induced decrease of p-MARCKS in SUM190 only. 

### 3.5. Inhibition of MARCKS and Its Phosphorylated Form by MPS Upregulates PTEN and Downregulates AKT Pathway in IBC Cells 

Using Western blot analysis, we assessed the total and phosphorylated AKT expression levels in both cell lines in untreated and treated conditions. We did not see any treatment-induced difference in total AKT expression in IBC and a slight decrease in nIBC cells. However, p-AKT was downregulated in SUM149 (Figure 2B), but was upregulated in MDA-MB-231 cells. We observed an upregulation of PTEN after MPS treatment in IBC cells only (Figure 2B). Finally, we evaluated if this PTEN upregulation affected the apoptotic process in SUM149 and MDA-MB-231 cells. In line with our results, cleaved caspase 3 were upregulated after MPS treatment only in SUM149 cells (Figure 2B). In addition, PTEN and cleaved PARP were upregulated and p-AKT was downregulated after MPS treatment in SUM190 cells, whereas no effect (pAKT) or an opposite effect (PTEN, cleaved PARP) were found in MCF7 cells (Figure S2F).

### 3.6. MARCKS and PTEN Protein Expressions Correlate with Survival in Patients with IBC, but Not in Patients with nIBC

**Figure 3:**
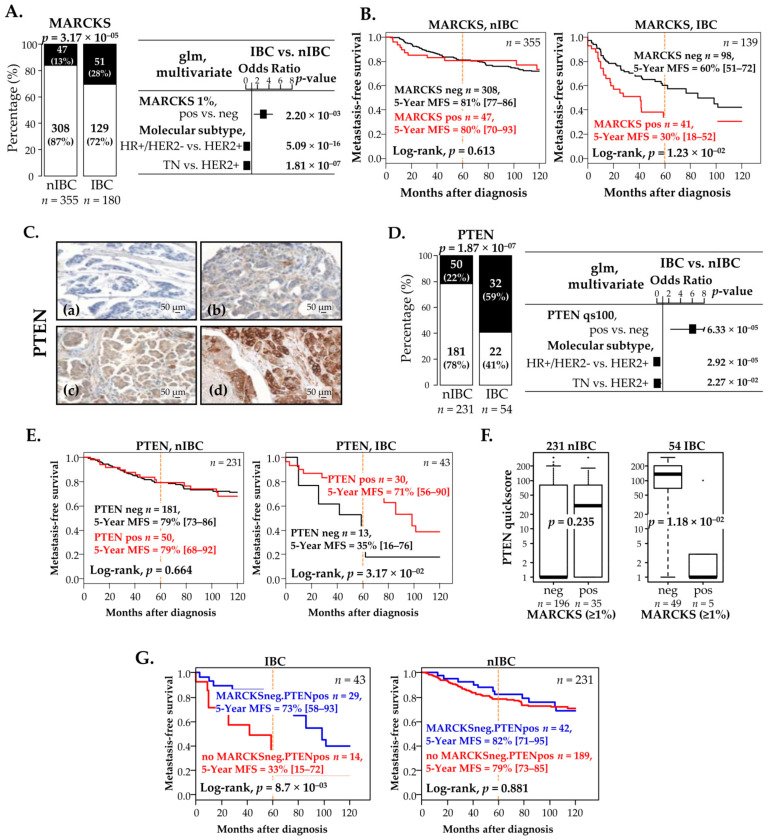
Protein expression of MARCKS and PTEN in IBC and nIBC and prognostic value. (**A**) Left: protein expression of MARCKS in IBC and nIBC patients (white: negative expression; black: positive expression). Right: forest plots showing the Odds Ratio (log10) of MARCKS expression level in IBC vs. nIBC group in a multivariate logistic regression analysis along with molecular subtypes. (**B**) Kaplan-Meier MFS curves in nIBC and IBC patients according to MARCKS expression (black: negative; red: positive). (**C**) Immunohistochemistry staining of PTEN expression in IBC vs. nIBC samples. The illustrated images are represented in; (a) a negative expression, (b) a 20% of staining, (c) 70% of staining, and (d) a 100% of staining: the staining was mainly cytoplasm and some nuclear, and 20× as magnification. (**D**) Similar to (**A**), but for PTEN expression. (**E**) Similar to (**B**), but for PTEN expression. (**F**) Box plots of PTEN expression (quick score) according to MARCKS expression group in nIBC (left) and IBC (right) samples, showing the negative correlation in IBC only. (**G**) Similar to (**B**) but for the combined expression of MARCKS and PTEN (blue: MARCKS-negative/PTEN-positive; red: no MARCKS-negative/PTEN-positive).

We analyzed MARCKS expression in a series including 180 IBC and 355 nIBC clinical samples. Their characteristics are summarized in Table 1. Using the same cut-off as previously defined (≥1%) [7], 28% of IBC samples vs. 13% of nIBC samples were defined as MARCKS-positive: this difference was significant (*p* = 3.17 × 10^−5^), even after adjustment upon the molecular subtypes (*p* = 2.20 × 10^−3^, Figure 3A) and upon all clinicopathological variables (Figure S4).

PositiveMARCKS expression was not associated with any tested clinicopathological feature in IBC (age, pathological type and grade, and molecular subtypes) but was associated with higher pathological grade and more aggressive molecular subtypes in nIBC (Table 1). Indeed, the percentage of HER2+ patients was 4-time higher in the MARCKS-positive vs. MARCKS-negative nIBC patients (20% vs. 5%), whereas it was similar in the MARCKS-positive vs. MARCKS-negative IBC patients (45% vs. 49%, respectively); similarly, the percentage of TN patients was 3.5-time higher in the MARCKS-positive vs. MARCKS-negative nIBC patients (37% vs. 11%), whereas it was 2-time higher in the MARCKS-positive vs. MARCKS-negative IBC patients (21% vs. 9%, respectively). More interestingly, MARCKS expression displayed a prognostic value for MFS in patients with IBC but not in patients with nIBC (Figure 3B, Table S2). Indeed, among the 139 IBC informative patients and with a median follow-up of 42 months (range, 1–160), the 5-year MFS was 30% (95 CI 18–52) in the MARCKS-positive group vs. 60% (95 CI 51–72) in the MARCKS-negative group (*p* = 1.23 × 10^−1^, log-rank test). The respective median follow-up in each group were 38 and 50 months, and the respective numbers of events were 21 (55%) and 41 (43%). Such unfavorable prognostic value was maintained in multivariate analysis (*p* = 3.13 × 10^−1^; Table S2). By contrast, among the 355 nIBC informative patients and with a median follow-up of 91 months (range, 4–231), MARCKS did not have any prognostic effect with a 5-year MFS of 80% (95 CI 70–93) in the MARCKS-positive group vs. 81% (95 CI 77–86) in the MARCKS-negative group (*p* = 0.613, log-rank test). The respective median follow-up in each group were 101 and 101 months, and the respective numbers of events were 11 (23%) and 83 (27%). In a prognostic analysis *per* molecular subtype (Figure S5), the MARCKS-positive group showed shorter MFS than the MARCKS-negative group in each molecular subtype in IBC, significantly in the HER2+ subtype (*p* = 4.92 × 10^− 1^, log-rank test) and non-significantly in the HR+/HER2– and TN subtypes in which the number of patients was small (*n* = 39 and 14, respectively). By contrast, no prognostic value was evidenced in nIBC in the three molecular subtypes.

The same analysis was carried out with PTEN IHC. Unfortunately, PTEN staining was assessable in 54 IBC samples and 231 nIBC samples only (Figure 3C). Figure S6 shows different intensities of Quick Score in IBC and nIBC samples. In this case, 59% of IBC samples vs. 22% of nIBC samples were identified as PTEN-positive (*p* = 1.87 × 10^−1^; Figure 3D), and the difference remained significant after adjustment upon the molecular subtypes (*p* = 6.33 × 10^− 1^). PTEN expression was not associated with any tested clinicopathological feature in both IBC and nIBC (Table 2). Regarding prognosis, PTEN expression was associated with longer MFS in patients with IBC but not in nIBC (Figure 3E, Table S3). Among the 43 informative IBC patients and with a median follow-up of 58 months (range, 1–159), the 5-year MFS was 71% (95 CI 56–90) in the PTEN-positive group vs. 35% (95 CI 16–79) in the PTEN-negative group (*p* = 3.17 × 10^−1^, log-rank test). The respective median follow-up in each group were 70 and 28 months, and the respective numbers of events were 14 (48%) and 10 (77%). In multivariate analysis, PTEN tended to keep its favorable prognostic value (*p* = 8.31 × 10^−1^; Table S3). The median follow-up was 85 months (range, 4–231) among the 231 informative nIBC patients, and the 5-year MFS was 79% in both PTEN-positive (95 CI 68–92) and PTEN-negative (95 CI 73–86) groups (*p* = 0.664, log-rank test). The respective median follow-up in each group were 126 and 96 months, and the respective numbers of events were 16 (32%) and 48 (27%).

### 3.7. Negative Correlation between MARCKS and PTEN Protein Expression in IBC Patients and Prognostic Value

Given the upregulation of PTEN after MPS-based MARCKS inhibition in IBC and our prognostic results, we analyzed the correlation between MARCKS (discrete value) and PTEN protein expression in the informative samples (54 IBC and 231 nIBC). Analysis of PTEN as continuous value (Quick Score) found a negative correlation within IBC samples (*p* = 1.18 × 10^−1^, Mann-Whitney test), but no correlation within nIBC samples (*p* = 0.235; Figure 3F). When PTEN was analyzed as discrete value (Quick Score), there was a trend for negative correlation among IBC samples (*p* = 9.53 × 10^−1^; logit link test), but no correlation amongn IBC samples (*p* = 0.85; data not shown). Patients with MARCKS-positive IBC displayed smaller PTEN expression than patients with MARCKS-negative IBC. Given their opposite prognostic value in IBC, we searched for an eventual prognostic complementarity between MARCKS and PTEN protein expressions. Among the patients with IBC, the “MARCKS-negative/PTEN-positive” group displayed 73% 5-year MFS (95 CI 58–93) whereas the remaining group including all other patients (MARCKS-negative/PTEN-negative, MARCKS-positive/PTEN-positive, and MARCKS-positive/PTEN-negative) thereafter named “no MARCKS-negative/PTEN-positive” group, displayed 33% 5-year MFS (95 CI 15–72; *p* = 8.70 × 10^−1^; Figure 3G, Table S4). Interestingly this prognostic value was maintained in multivariate analysis (*p* = 2.81 × 10^−1^; Table S4). By contrast, the 2-protein model was not prognostic in patients with nIBC (Figure 3G), with 82% 5-year MFS (95 CI 71–95) in the “MARCKS-negative/PTEN-positive” group and 79% 5-year MFS (95 CI 73–85) in the “no MARCKS-negative/PTEN-positive” group.

## 4. Discussion

In the present study, we tested in vitro the effects of MARCKS inhibition by treating IBC vs. nIBC cells with MPS in terms of cell proliferation, migration and invasion, mammosphere formation, and signaling pathways. We then tried to expand these in vitro findings on IBC vs. nIBC clinical tumor samples at the protein level using IHC. To our knowledge, this is the first study to evaluate the functional consequences of MARCKS inhibition in IBC vs. nIBC cell lines.

MPS treatment significantly decreased the proliferation and colony formation rates of IBC cells, whereas it did not affect those of nIBC cells. Furthermore, invasion and migration were also significantly impaired after MPS treatment in IBC cells but were not affected in nIBC cells. These results suggest a functional role of MARCKS in IBC aggressiveness, which is consistent with the poor-prognosis value of its expression in IBC patients. These results are also consistent with the literature that reports the role of MARCKS in promoting cancer cell proliferation [22,23,24] or invasion and migration [25,26,27]. All these effects were observed in our IBC cell lines. More indirectly, our IHC results show the overexpression of MARCKS in cancer cells located in invasive margins in 18 out of 19 tested samples and in tumor emboli. Furthermore, our transcriptomics analyses shows the co-expression of EMT markers with *MARCKS* expression in our IBC clinical samples to further suggest a potential role in aggressiveness. One important characteristic of IBC cells is their known association with tumor stemness gene signatures and their ability to self-renew, growing as “mammospheres” in 3D culture [18,28,29]. We showed that MARCKS inhibition only impaired the primary mammosphere formation in IBC cells. Since the mammosphere formation may depend on E-cadherin expression [30], flow cytometry deserves to be applied to analyze stemness markers in our BC cell lines before and after MPS treatment. 

**Figure 4:**
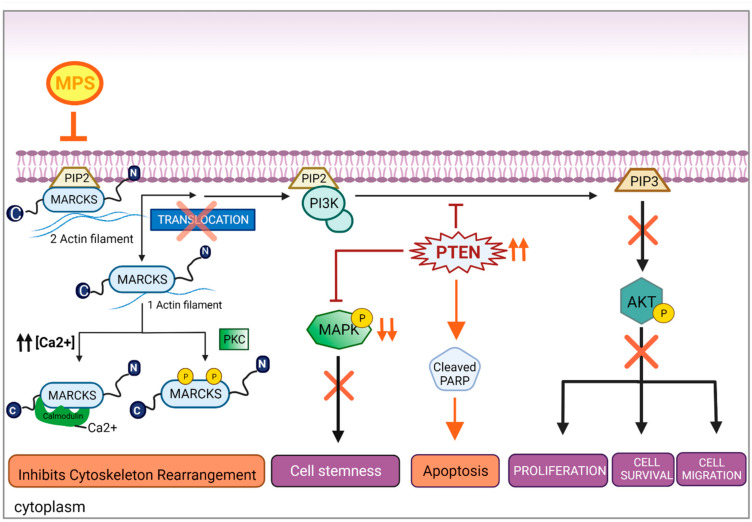
MPS peptide specifically suppressed the activation of MARCKS and regulated PTEN/AKT and MAPK pathways in inflammatory breast cancer cells. MARCKS is involved in various cellular processes, particularly in the cytoskeleton control (by phosphorylation of MARCKS), proliferation, cell motility, and cell survival (by the PI3K/AKT pathway). Exposure of PIP2 on the membrane allows PI3K to phosphorylate it into PIP3, activating AKT-mediated signaling. However, this pathway is regulated by various factors, including PTEN. Our representative figure explains in part the mechanistic role of MPS peptide in IBC cells explaining how MARCKS inhibition subsequently inhibited AKT phosphorylation (represented by an orange cross and down arrows) and, more importantly, the upregulation of PTEN (represented by anascending arrows), leading to apoptosis and the downregulation of the MAPK pathway (represented by an orange cross and down arrows). These found regulations potentially led to the cytoskeleton rearrangement, proliferation, cell motility, tumor stemness inhibition, and apoptosis activation.

Our Western blot analysis showed that MARCKS inhibition regulated the PTEN/AKT and MAPK signaling pathways in IBC cells but not in nIBC cells. Figure 4 proposes a schema linking the MPS effects on cell signaling and cell behavior in IBC cells. The PTEN/PI3K/Akt signaling pathway plays a key role in BC’s progression, and the PI3K/Akt pathway is associated with cell motility and actin reorganization in IBC [17]. Moreover, MARCKS is known to activate the downstream PI3K/AKT pathway in cancer [31,32]. Indeed, several studies showed the key function of activated MARCKS in providing PIP2 that remains accessible to PI3K and form PIP3, which in its turn will phosphorylate AKT, thus inducing AKT pathway activation known to be responsible for activating extracellular matrix (ECM) proteins, cell proliferation, invasion, migration, and actin remodeling in different cancers [31,33,34,35,36]. Here, we showed that MARCKS inhibition decreased the AKT signaling pathway and increased the PTEN expression in IBC cells but not in nIBC. These results are consistent with two studies from Dr.Van Golen’s team. The Lehman et al.’s study [37] showed in vitro and in silico that the deregulation of the PI3K/AKT pathway in IBC was crucial for cell motility and invasion and had a role in cell survival, which was not found in nIBC cells and patients. Our results suggest that MARCKS, overexpressed in IBC, might be responsible for such PI3K/AKTderegulation. In the second study by Van Golen et al. [17], the authors identified RhoC as overexpressed in IBC vs. nIBC; this protein is involved in cytoskeleton organization and cell invasion/migration, and is known as one of the major activators of MARCKS protein. These findings strengthen our findings that show how MARCKS, a key protein regulating cytoskeleton remolding, deregulates the PTEN/AKT pathway in IBC cells.That might partly explain why IBC cells are more aggressive than nIBC cells and why MARCKS overexpression is associated with poor MFS in IBC. Regarding MAPK, some studies demonstrated that the activated MAPK pathway is responsible for IBC aggressiveness, promoting proliferation, stemness, and metastatic potential [17,38,39]. In this study, we showed that targeting MARCKS decreased phosphorylated-MAPK protein expression only in IBC cells. These results are coherent with the literature by the specific stemness of IBC cells and by the role of MAPK dysregulation in increasing the cell stemness in IBC. However, from the literature, MAPK was found to be an alternative activator of MARCKS through RhoC/MAPK pathway, inducing MARCKS phosphorylation responsible for cell aggressiveness and cytoskeleton remodeling. In a study on breast cancer, authors showed that PTEN regulated MAPK and that Pten loss should be maintained to keep upregulating PI3K and MAPK cascades, resulting in tumor progression [40]. We suggest that MARCKS inhibition in IBC cells regulates MAPK as a downstream signaling pathway through PTEN upregulation, which could partially explain why the mammosphere formation was impaired by MPS treatment only in IBC cells.

As a next step and given the IBC-specific negative correlation between MARCKS and PTEN after MPS treatment, we evaluated MARCKS and PTEN protein expression in our series of clinical IBC and nIBC samples. First, we confirmed the overexpression of MARCKS in IBC vs. nIBC independently from the molecular subtypes and all other clinicopathological variables tested. Interestingly, positive MARCKS expression showed correlations with clinicopathological features, including MFS, which differed between IBC and nIBC. In IBC, no association existed with the classical prognostic variables, but a strong and independent association was observed with shorter MFS. In nIBC, the opposite was found with correlation with unfavorable prognostic variables (grade and molecular subtypes), but no association with MFS. This conundrum warrants further investigation. We do not think that this prognostic divergence is related to a low statistical power for several reasons: (i) the number of IBC patients was lower than the number of nIBC patients, (ii) the number of events was relatively important in each population (33% in IBC and 21% in nIBC), and (iii) the follow-up was longer in nIBC than IBC (91 vs. 42 months). We suppose that it might be related to different modes of metastatic dissemination between IBC and nIBC, in which MARCKS might be involved. PTEN overexpression was more frequent in IBC than nIBC but was associated with longer MFS in IBC only. Second, we found a negative correlation: between MARCKS and PTEN expressions in IBC samples, whereas no correlation (negative or positive) was evidenced in nIBC samples. Finally, since MARCKS inhibition leads to PTEN upregulation in IBC cells and given the opposite prognostic value of both proteins in IBC, we searched for an eventual prognostic complementarity between MARCKS and PTEN protein: we found that the group “MARCKS-negative/PTEN-positive” displayed longer 5-year MFS (73%) than the remaining group “no MARCKS-negative/PTEN-positive” (33% 5-year MFS). Such correlation was observed in IBC patients only. Interestingly, this IHC profile with a good-prognosis value mirrored the molecular profile (MARCKS-downregulated/PTEN-upregulated) of MPS-treated IBC cell lines, indirectly the potential therapeutic benefit of MARCKS inhibition. 

Thus, our results suggest a functional role of MARCKS in IBC aggressiveness by affecting cell proliferation, migration and invasion, mammosphere formation, and apoptosis and how its inhibition regulates different downstream pathways in IBC. These findings confirm the published data regarding the specific association of PTEN/PI3K/AKT pathway with IBC compared to nIBC and how its deregulation is required for IBC invasiveness and cell motility. Analysis of clinical samples suggests the independent poor-prognosis value of MARCKS expression in IBC. By contrast, the MARCKS inhibitor (MPS) did not affect the two nIBC cell lines, and MARCKS expression had no prognostic value in patients with nIBC. Such divergence between IBC and nIBC samples might suggest an IBC-specificity regarding the role of MARCKS, even if we cannot exclude a role in nIBC. However, our study displays several limitations. Regarding the pre-clinical data, the number of BC cell lines remains limited (2 IBC and 2 nIBC) and does not allow a complete representation of all molecular subtypes; analysis of more cell lines of each molecular subtype is warranted, as well as the deployment and analysis of in vivo animal models that are better adapted than cell lines to investigate the aggressiveness and metastatic properties related to MARCKS. Regarding the clinical data, their retrospective nature is a limitation as well as its associated biases, such as the variable number of samples informative for each clinicopathological variable; even if IBC is a rare disease, the number of clinical samples remains relatively limited and does not allow an analysis per molecular subtype. Another limitation was the limited number of patients with both MARCKS and PTEN data. Clearly, analysis of more pre-clinical models and clinical samples is warranted. IHC analysis on standard slides of a large series of IBC and nIBC clinical samples would allow analyzing expression of MARCKS and EMT proteins. In addition, multiplex immunofluorescence would be suitable for assessing the co-expression of MARCKS and EMT proteins on cancer cells. For both analyses, expression and co-expression in the whole tumor but also in invasive margins deserve to be assessed, as well as eventual correlations with clinical outcome. However, the overexpression of MARCKS in IBC, as well as IBC functional consequences of its inhibition, suggest that MARCKS could become a therapeutic target of IBC and deserves further evaluation. 

## 5. Conclusions

In vitro, we showed that MARCKS inhibition impaired the cell proliferation, invasion, migration, and mammosphere formation, and regulated the PTEN/AKT and MAPK signaling pathways in IBC cells but not in nIBC cells. Analysis of clinical samples by IHC showed that MARCKS-negative/PTEN-positive protein expression was associated with longer MFS in patients with IBC only. These results suggest that MARCKS is a new potential therapeutic target in the 28% of patients with MARCKS-positive IBC. Due to the above-cited limitations of our study, further in vitro and in vivo studies are required to validate the role of MARCKS; if validated, the testing of MARCKS inhibitors in IBC pre-clinical models is warranted in this mysterious and so lethal disease.

## Figures and Tables

**Table 1:** Correlations of MARCKS expression with clinicopathological variables in 180 IBC and 355 nIBC.

Characteristics		*n*	Global	MARCKS, IBC		*p*-Value	*n*	Global	MARCKS, nIBC		*p*-Value
				Negative	Positive				Negative	Positive	
Age		145	50 (15–81)	51 (27–80)	51 (15–81)	0.966	355	58 (25–94)	59 (25–94)	58 (37–87)	0.642
Histology						0.795					0.126
	ductal	151	151 (89%)	108 (90%)	43 (88%)		247	247 (70%)	216 (70%)	31 (66%)	
	lobular	13	13 (8%)	8 (7%)	5 (10%)		47	47 (13%)	41 (13%)	6 (13%)	
	mixed	1	1 (1%)	1 (1%)	0 (0%)		16	16 (5%)	16 (5%)	0 (0%)	
	other	4	4 (2%)	3 (2%)	1 (2%)		45	45 (13%)	35 (11%)	10 (21%)	
Grade						0.460				<1.0 × 10^−5^	
	1	10	10 (6%)	6 (5%)	4 (9%)		119	119 (34%)	111 (36%)	8 (17%)	
	2	52	52 (32%)	40 (35%)	12 (26%)		144	144 (41%)	131 (43%)	13 (28%)	
	3	99	99 (61%)	69 (60%)	30 (65%)		91	91 (26%)	66 (21%)	25 (54%)	
pT											0.091
	pT1	---	---	---	---		147	147 (41%)	130 (42%)	17 (36%)	
	pT2	---	---	---	---		149	149 (42%)	123 (40%)	26 (55%)	
	pT3	---	---	---	---		59	59 (17%)	55 (18%)	4 (9%)	
pN											1.000
	0	---	---	---	---		177	177 (50%)	154 (50%)	23 (50%)	
	1	---	---	---	---		175	175 (50%)	152 (50%)	23 (50%)	
Molecular subtype						0.204				2.04 × 10^−8^	
	HER2+	54	54 (47%)	37 (49%)	17 (45%)		22	22 (7%)	14 (5%)	8 (20%)	
	HR+/HER2−	45	45 (39%)	32 (42%)	13 (34%)		250	250 (79%)	232 (84%)	18 (44%)	
	TN	15	15 (13%)	7 (9%)	8 (21%)		44	44 (14%)	29 (11%)	15 (37%)	
Follow-up median, months (range)		139	42 (1–160)	43 (1–159)	23 (1–160)	0.777	355	91 (4–231)	90 (4–231)	101 (5–216)	0.698
MFS event, *n*(%)		139	68 (33%)	44 (45%)	24 (59%)	0.193	355	94 (21%)	83 (27%)	11 (23%)	0.724
5-year MFS [95%CI]		139	52% [43–62]	60% [51–72]	30% [18–52]	1.23× 10^−2^	355	81% [77–85]	81% [77–86]	80% [70–93]	0.613

**Table 2:** Correlations of PTEN expression with clinicopathological variables in 54 IBC and 231 nIBC.

Characteristics		*n*	Global	PTEN, IBC		*p*-Value	*n*	Global	PTEN, nIBC		*p*-Value
				Negative	Positive				Negative	Positive	
Age		32	55 (33.25)	53 (33–80)	57 (37–78)	0.385	231	58 (25–94)	60 (25–94)	57 (35–87)	0.125
Histology						0.235					0.941
	ductal	46	46 (90%)	17 (85%)	29 (94%)		172	172 (74%)	134 (74%)	38 (76%)	
	lobular	4	4 (8%)	3 (15%)	1 (3%)		21	21 (9%)	17 (9%)	4 (8%)	
	mixed	1	1 (2%)	0 (0%)	1 (3%)		7	7 (3%)	5 (3%)	2 (4%)	
	other	0	0 (0%)	0 (0%)	0 (0%)		31	31 (13%)	25 (14%)	6 (12%)	
Grade						0.229					0.239
	1	3	3 (6%)	0 (0%)	3 (10%)		67	67 (29%)	56 (31%)	11 (22%)	
	2	13	13 (25%)	4 (20%)	9 (29%)		98	98 (43%)	72 (40%)	26 (53%)	
	3	35	35 (69%)	16 (80%)	19 (61%)		65	65 (28%)	53 (29%)	12 (24%)	
pT											0.143
	pT1	---	---	---	---		88	88 (38%)	72 (40%)	16 (32%)	
	pT2	---	---	---	---		104	104 (45%)	83 (46%)	21 (42%)	
	pT3	---	---	---	---		39	39 (17%)	26 (14%)	13 (26%)	
pN											0.759
	0	---	---	---	---		113	113 (49%)	90 (50%)	23 (46%)	
	1	---	---	---	---		118	118 (51%)	91 (50%)	27 (54%)	
Molecular subtype						0.962					0.571
	HER2+	12	12 (43%)	5 (45%)	7 (41%)		19	19 (9%)	15 (9%)	4 (9%)	
	HR+/HER2−	13	13 (46%)	5 (45%)	8 (47%)		165	165 (77%)	129 (76%)	36 (82%)	
	TN	3	3 (11%)	1 (9%)	2 (12%)		30	30 (14%)	26 (15%)	4 (9%)	
Follow-up median, months (range)		43	58 (1–159)	35 (9–159)	67 (1–143)	0.223	231	85 (4–231)	82 (4–206)	104 (5–231)	3.35 × 10^−2^
MFS event, *n*(%)		43	25 (37%)	10 (77%)	14 (48%)	0.104	231	64 (22%)	48 (27%)	16 (32%)	0.477
5-year MFS [95%CI]		43	60% [46–78]	35% [16–76]	73% [58–93]	1.85 × 10^−2^	231	79% [74–85]	79% [73–86]	79% [68–92]	0.664

## Data Availability

Data are available upon reasonable request.

## Supplementary Materials

The following supporting information can be downloaded at: https://www.mdpi.com/article/10.3390/cells11182926/s1; Figure S1: MARCKS promotes cell proliferation and motility of IBC; Figure S2: MARCKS promotes cell proliferation, motility and mammosphere formation in IBC; Figure S3: MARCKS protein overexpression in invasive margins and emboli in IBC clinical tumor samples; Figure S4: Uni- and multivariate analyses of IBC/nIBC distinction. Figure S5: Prognostic analysis of MARCKS expression in IBC and nIBC in each molecular subtype separately; Figure S6: PTEN immunostaining in clinical breast cancer samples; Table S1:Multivariate analysis of correlation between MARCKS expression (positive vs. negative) and expression of genes and signatures/scores related to EMT and stemness in IBC clinical samples; Table S2: Uni- and multivariate prognostic analysis of MFS in patients with IBC and patients with nIBC, according to MARCKS IHC and clinicopathological variables; Table S3: Uni- and multivariate prognostic analysis of MFS in patients with IBC and patients with nIBC, according to PTEN IHC and clinicopathological variables. Table S4: Uni- and multivariate prognostic analysis of MFS in patients with IBC and patients with nIBC, according to MARCKS/PTEN IHC combination and clinicopathological variables.
