# Supplementary material for: MARCKS as a Potential Therapeutic Target in Inflammatory Breast Cancer
Source: Cells. 2022 Sep 19;11(18):2926. doi: 10.3390/cells11182926 (PMC9496908; doi:10.3390/cells11182926)
Supplement: Supplementary file 1 [file cells-11-02926-s001.zip › cells-1832917-supplementary.pdf]

Supplementary Material

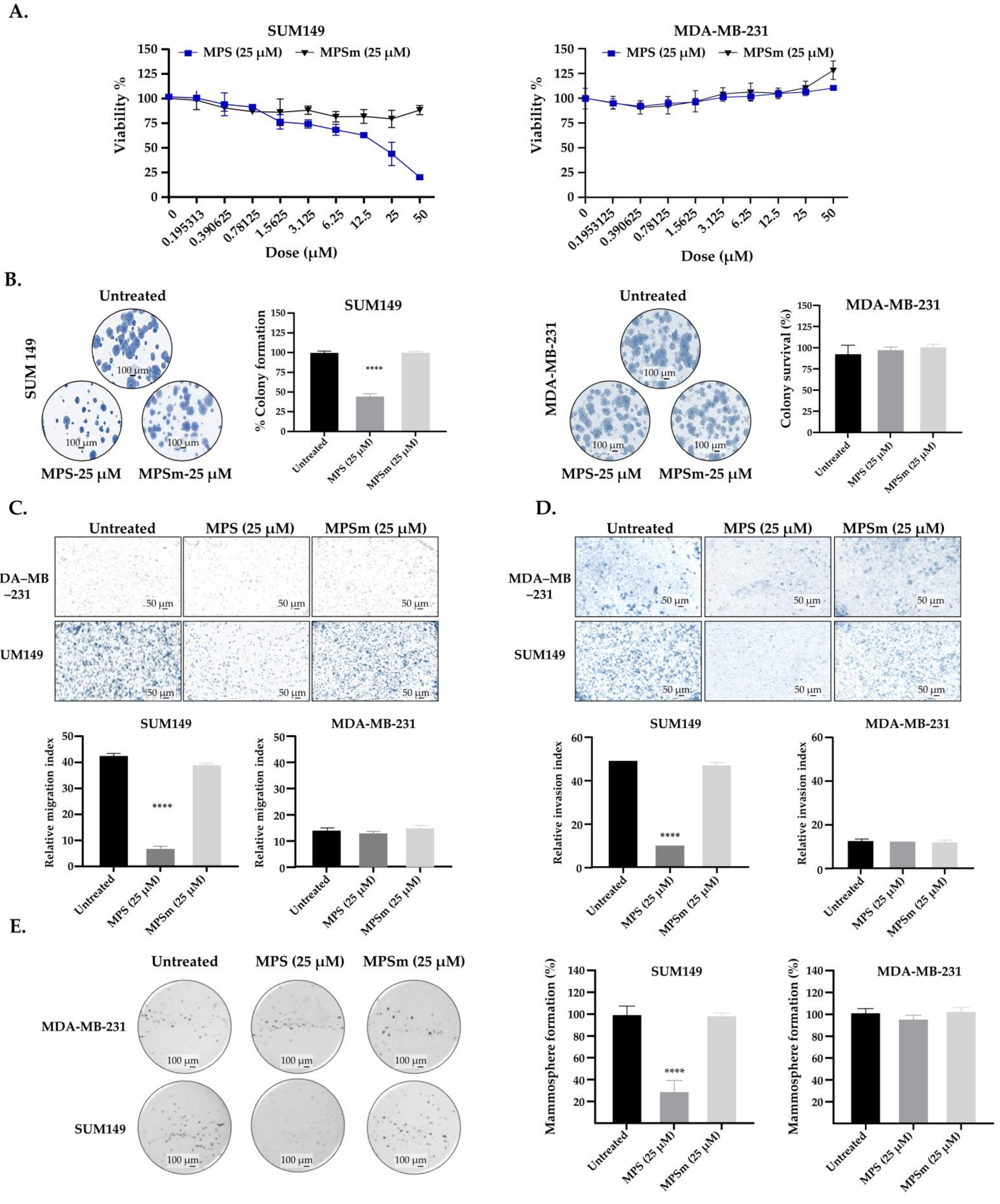

Figure S1. MARCKS promotes cell proliferation and motility of IBC. (**A**–**E**) Same as figures 1 and 2A, but we added the data for cells treated with MPS mutated or scrambled (MPSm) as a control for all our in vitro experiments. As shown in figures (**A**) (proliferation assay), (**B**) (colony formation assay), (**C**) (migration assay in chamber), (**D**) (invasion assay in chamber with Matrigel), and (**E**) (mammosphere formation assay), all the different experiments did not show any significant difference in terms of proliferation, cell motility and mammosphere formation between the untreated cells and the treated cells with MPSm. Data were represented as mean ± SD. ****$p \leq 0.0001$, (3 replicates).

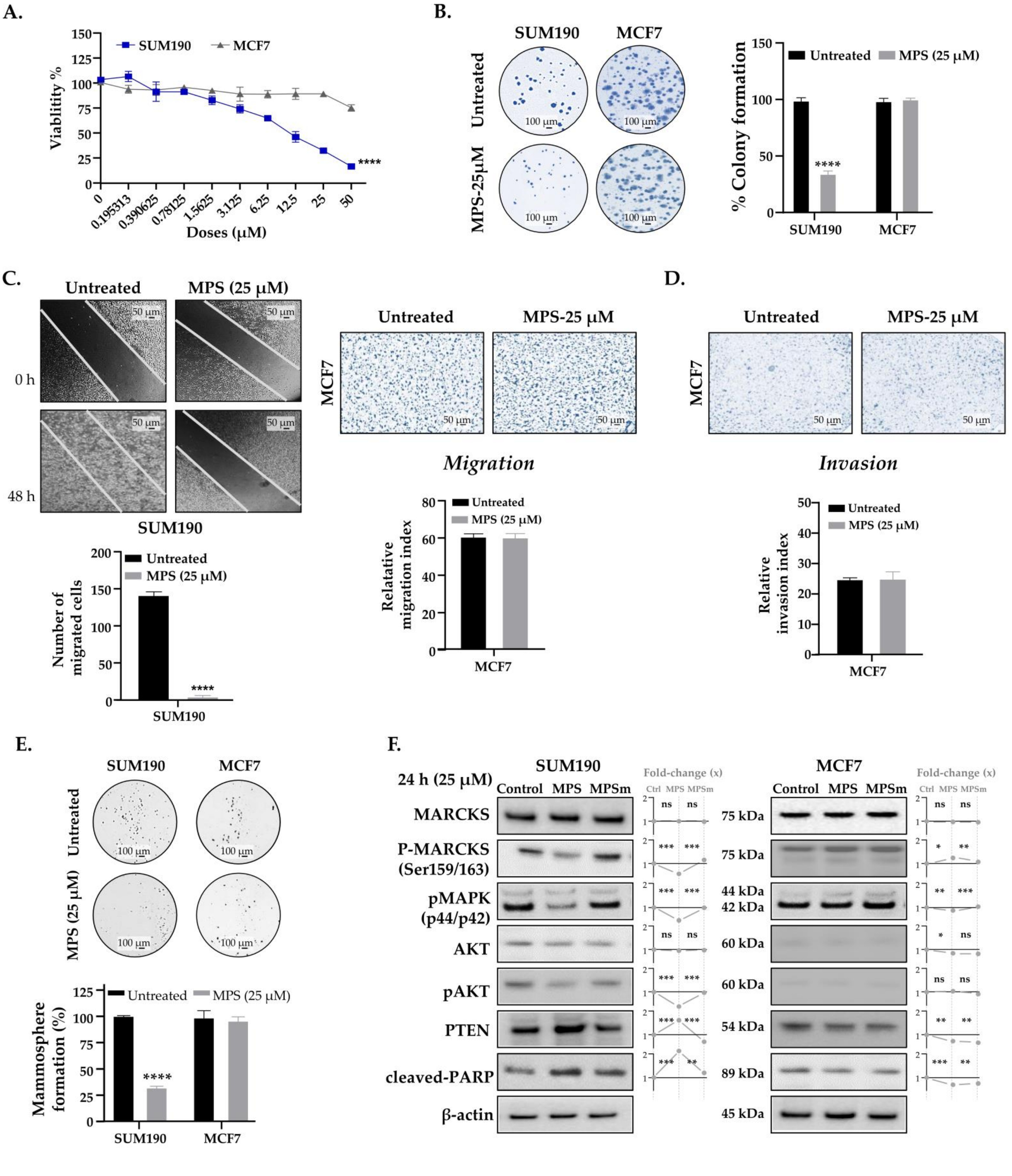

Figure S2. MARCKS promotes cell proliferation, motility, and mammosphere formation in IBC. (**A**–**F**). Same as figures 1 and 2 but for two other breast cancer cell lines overexpressing MARCKS: the SUM190 IBC cell line and the MCF7 nIBC cell line: (**A**) (proliferation assay), (**B**) (colony formation assay), (**C**) (migration assay in chamber (MCF7) and in scratch/wound-healing assay (SUM190)), (**D**) (invasion assay in chamber with Matrigel (MCF7)). Because SUM190 are big cells, we could not realize the migration and invasion in transwell chambers. (**E**) (mammosphere formation assay), and (**F**) (Western blot analysis). All the different experiments validated the results observed with SUM149 and MD-MB-231. Data were represented as mean ± SD. ****$p \leq 0.0001$, ***$p \leq 0.001$, **$p \leq 0.01$, *$p \leq 0.05$, ns: $p > 0.05$, (3 replicates).

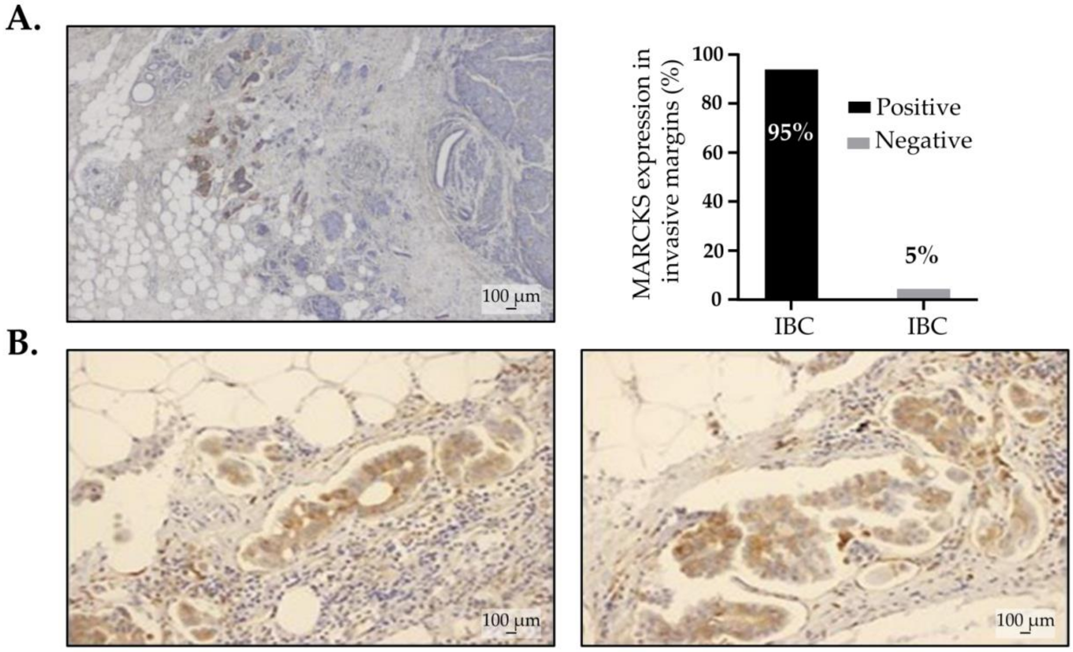

Figure S3. MARCKS overexpression in invasive margins and tumor emboli in IBC clinical tumor samples. (**A**) Left: IHC images showing MARCKS-positive cancer cells more expressed in the invasive margins (magnification ×10); right: Box plot showing the percentage of MARCKS-positive IBC tumor samples with (black) and without (grey) positive cancer cells in the invasive margins. (**B**) IHC images of MARCKS-positive cancer cells within tumor emboli for two different patients. (magnification ×20).

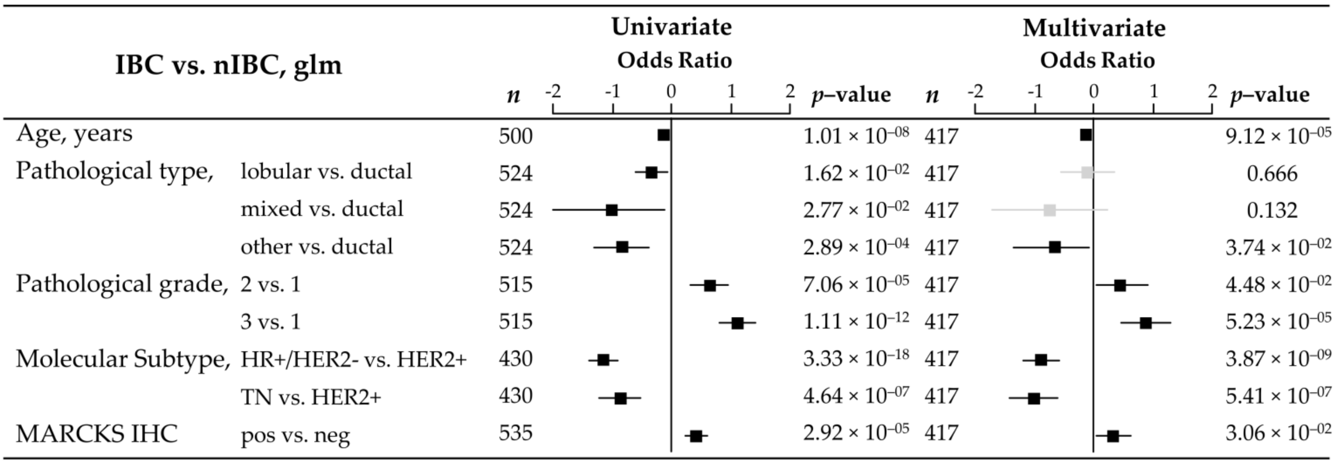

Figure S4. Uni- and multivariate analyses of IBC/nIBC distinction. Forest plots showing the Odds Ratio (log10) of MARCKS expression level in IBC vs. nIBC group in a multivariate logistic regression analysis along with all clinicopathological variables.

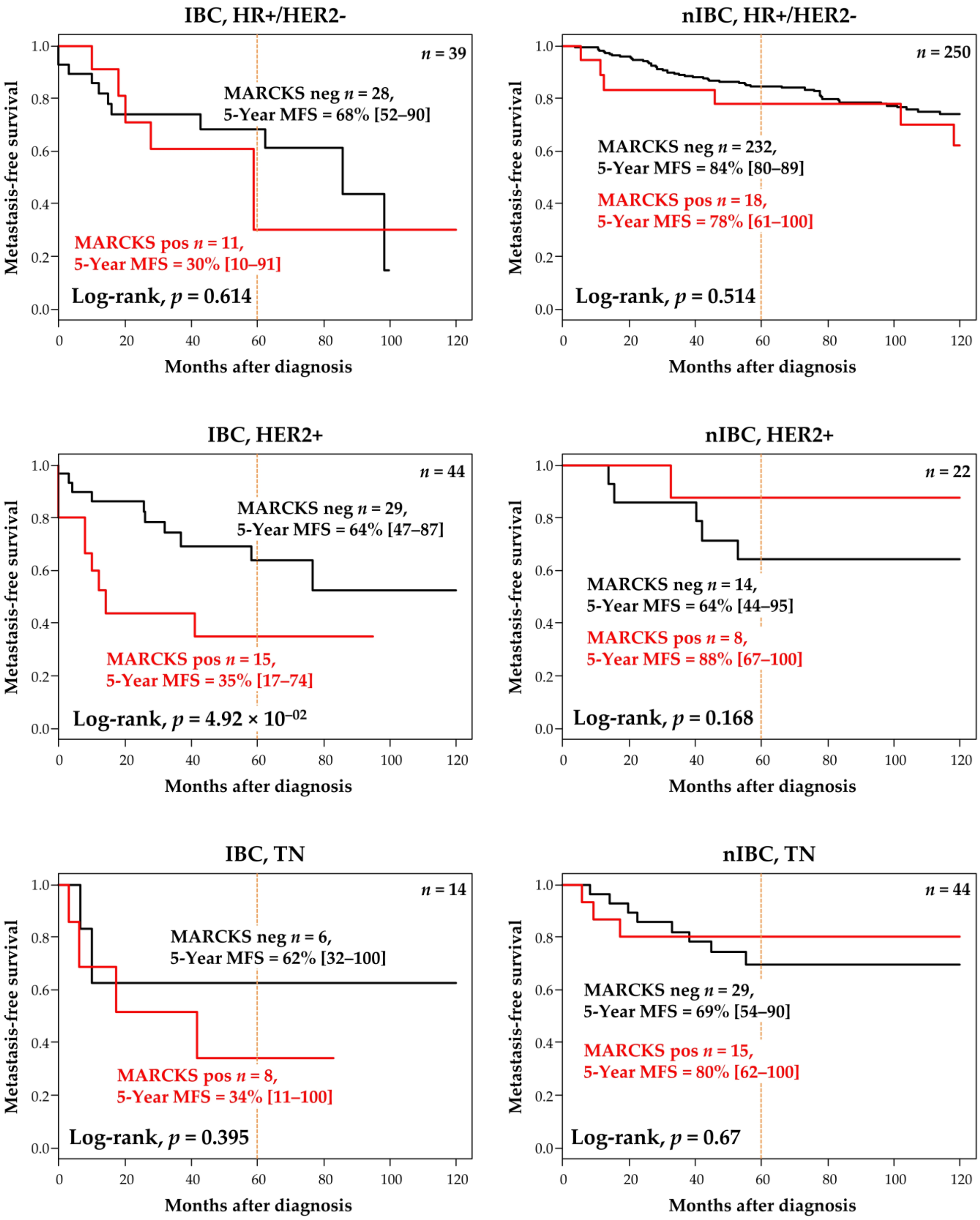

Figure S5. Prognostic analysis of MARCKS expression in IBC and nIBC in each molecular subtype separately. Kaplan-Meier MFS curves in IBC (left) and nIBC (right) patients according to MARCKS expression (black: negative; red: positive) in the HR+/HER2– subtype (top), in the HER2+ subtype (middle), and in the TN subtype (bottom).

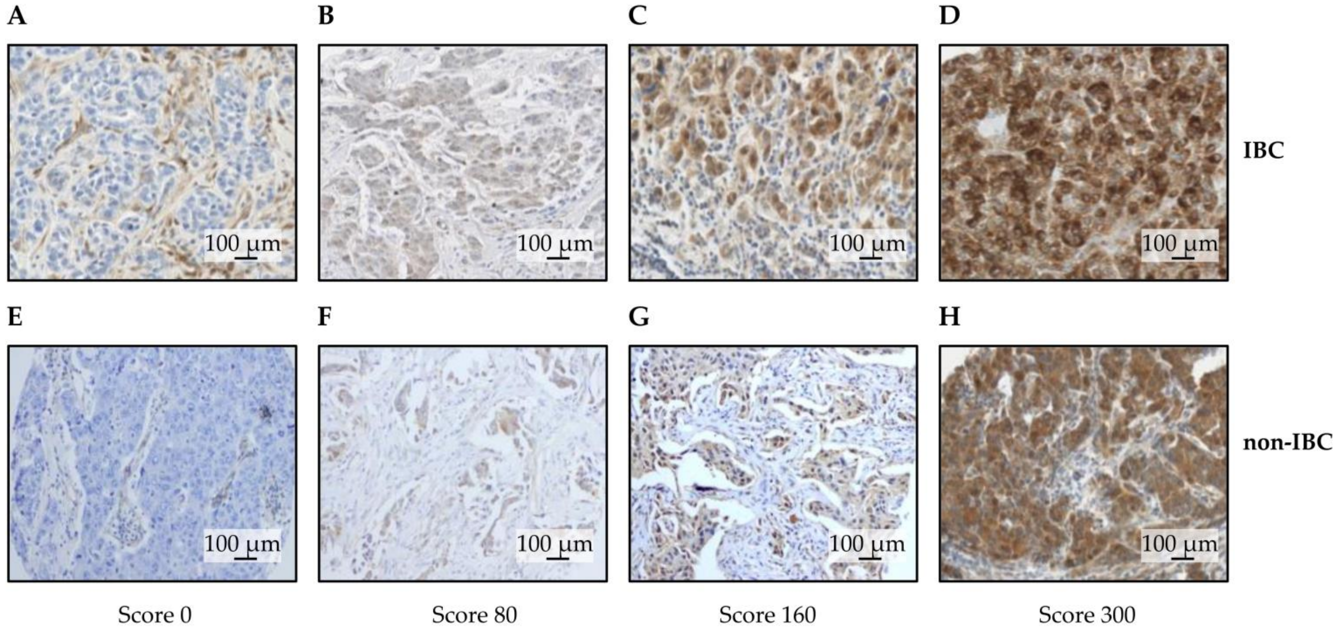

Figure S6. PTEN immunostaining in clinical breast cancer samples.**(A–D**) Representative images of immunohistochemistry staining in IBC samples show the different scores of expression: score 0 (**A**), score 80 (**B**), score 160 (**C**), and score 300 (**D**) (quick-score = percentage × intensity) (magnification ×20). (**E**–**H**) Representative images of IHC staining in nIBC samples: (**E**) shows a score of 0, (**F**) score 80, (**G**) score 160, and (**H**) score 300.

Table S1. Multivariate analysis of correlation between MARCKS expression (positive vs. negative) and expression of genes and signatures/scores related to EMT and stemness in IBC clinical samples.

	**Multivariate MARCKS ~**		**IBC**	
	**Variable + Molecular Subtypes**	***n***	**Odds Ratio (95%CI)**	***p*-Value***
EMT genes	*TWIST2* mRNA	71	1.33 (1.12–1.58)	1.89×10^{-03}
	ZEB1 mRNA	71	1.96 (1.65–2.33)	1.10×10^{-10}
	ZEB2 mRNA	71	1.86 (1.49–2.31)	4.74×10^{-07}
	TWIST1 mRNA	71	1.48 (1.25–1.75)	1.68×10^{-05}
	VIM mRNA	71	2.02 (1.69–2.42)	1.09×10^{-10}
	EMT metagene [16]	71	2.03 (1.47–2.80)	4.95×10^{-05}
Stemnessge nes	*ALDH1A1* mRNA	71	1.55 (1.28–1.87)	2.73×10^{-05}
	Mammary Stem Cell score [20]	71	1.97 (1.05–3.71)	3.90×10^{-02}
	Progenitor Luminal score [20]	71	0.67 (0.32–1.43)	0.303
	Mature Luminal score [20]	71	0.72 (0.33–1.56)	0.408
	CDH1 mRNA	71	0.99 (0.81–1.22)	0.948
	CD44+/*CD24*– vs. other	71	1.06 (0.77–1.47)	0.704

*, the *p*-value is for the logit link test.

Table S2. Uni- and multivariate prognostic analysis of MFS in patients with IBC and patients with nIBC, according to MARCKS IHC and clinico-pathological variables.

nIBC		*n*	Univariate MFS Odds Ratio (95%CI)	Univariate MFS *p*-Value	*n*	Multivariate MFS Odds Ratio (95%CI)	Multivariate MFS *p*-Value
Age, years		355	0.99 (0.98–1.01)	0.492			
Pathological type	lobular vs. ductal	355	1.43 (0.84–2.44)	2.15×10^{-02}	351	1.68 (0.97–2.93)	0.064
	mixed vs. ductal		1.59 (0.73–3.46)		351	1.52 (0.69–3.34)	0.298
	other vs. ductal		0.24 (0.07–0.75)		351	0.35 (0.11–1.12)	0.078
Pathological axillary node status, pN	1 vs. 0	352	3.08 (1.95–4.87)	1.46×10^{-06}	351	2.25 (1.40–3.63)	8.25×10-04
Pathological tumor size (pT)	pT2 vs. pT1	355	2.71 (1.62–4.53)	1.50×10^{-05}	351	1.78 (1.03–3.07)	3.72×10^{-02}
	pT3 vs. pT1		3.83 (2.14–6.85)		351	2.51 (1.38–4.56)	2.59×10^{-03}
Pathological grade	2 vs. 1	354	2.15 (1.24–3.73)	3.42×10^{-04}	351	1.62 (0.91–2.88)	0.100
	3 vs. 1		3.23 (1.82–5.75)		351	2.38 (1.27–4.44)	6.71×10^{-03}
Molecular Subtype	HR+/HER2–vs. HER2+	316	0.75 (0.34–1.64)	0.758			
	TN vs. HER2+		0.83 (0.32–2.13)				
MARCKS 1%	Pos vs. neg	355	0.85 (0.45–1.60)	0.614			

IBC		*n*	Univariate MFS Odds Ratio (95%CI)	Univariate MFS *p*-value	*n*	Multivariate MFS Odds Ratio (95%CI)	Multivariate MFS *p*-value
Age, years		115	1.02 (0.99–1.04)	0.173			
Pathological type	lobular vs. ductal	139	1.48 (0.67–3.25)	0.602			
	mixed vs. ductal		0.00 (0.00 - Inf)				
	other vs. ductal		0.40 (0.06–2.88)				
TNM, N	1 vs. 0	136	1.86 (1.03–3.36)	4.11×10^{-02}	136	1.89 (1.04–3.42)	3.70×10^{-02}
Pathological grade	2 vs. 1	135	0.63 (0.26–1.52)	0.538			
	3 vs. 1		0.80 (0.36–1.79)				
Molecular Subtype	HR+/HER2–vs. HER2+	97	1.04 (0.55–1.95)	0.992			
	TN vs. HER2+		1.04 (0.41–2.58)				
MARCKS 1%	Pos vs. neg	139	1.88 (1.14–3.11)	1.37×10^{-02}	136	1.89 (1.14–3.13)	3.13×10^{-02}

*, the *p*-value is for the Wald test.

Table S3. Uni- and multivariate prognostic analysis of MFS in patients with IBC and patients with nIBC, according to PTEN IHC and clinico-pathological variables.

nIBC		*n*	Univariate MFS Odds Ratio (95%CI)	*p*-Value	*n*	Multivariate MFS Odds Ratio (95%CI)	*p*-Value
Age, years		355	0.99 (0.98–1.01)	0.492			
Pathological type	lobular vs. ductal	355	1.43 (0.84–2.44)	2.15×10^{-02}	351	1.68 (0.97–2.93)	0.064
	mixed vs. ductal		1.59 (0.73–3.46)		351	1.52 (0.69–3.34)	0.298
	other vs. ductal		0.24 (0.07–0.75)		351	0.35 (0.11–1.12)	0.078
Pathological axillary node status, pN	1 vs. 0	352	3.08 (1.95–4.87)	1.46×10^{-06}	351	2.25 (1.40–3.63)	8.25×10^{-04}
Pathological tumor size (pT)	pT2 vs. pT1	355	2.71 (1.62–4.53)	1.50×10^{-05}	351	1.78 (1.03–3.07)	3.72×10^{-02}
	pT3 vs. pT1		3.83 (2.14–6.85)		351	2.51 (1.38–4.56)	2.59×10^{-03}
Pathological grade	2 vs. 1	354	2.15 (1.24–3.73)	3.42×10^{-04}	351	1.62 (0.91–2.88)	0.100
	3 vs. 1		3.23 (1.82–5.75)		351	2.38 (1.27–4.44)	6.71×10^{-03}
Molecular Subtype	HR+/HER2–vs. HER2+	316	0.75 (0.34–1.64)	0.758			
	TN vs. HER2+		0.83 (0.32–2.13)				
PTEN quick score-100	Pos vs. neg	231	1.13 (0.64–2.00)	0.665			

IBC		*n*	Univariate MFS Odds Ratio (95%CI)	*p*-Value	*n*	Multivariate MFS Odds Ratio (95%CI)	*p*-Value
Age, years		115	1.02 (0.99–1.04)	0.173			
Pathological type	lobular vs. ductal	139	1.48 (0.67–3.25)	0.602			
	mixed vs. ductal		0.00 (0.00–∞)				
	other vs. ductal		0.40 (0.06–2.88)				
TNM, N	1 vs. 0	136	1.86 (1.03–3.36)	4.11×10^{-02}	40	1.39 (0.56–3.43)	0.48
Pathological grade	2 vs. 1	135	0.63 (0.26–1.52)	0.538			
	3 vs. 1		0.80 (0.36–1.79)				
Molecular Subtype	HR+/HER2–vs. HER2+	97	1.04 (0.55–1.95)	0.992			
	TN vs. HER2+		1.04 (0.41–2.58)				
PTEN quick score-100	Pos vs. neg	43	0.42 (0.18–0.94]	3.56×10^{-02}	40	0.46 (0.19–1.11)	8.31×10^{-02}

*, the *p*-value is for the Wald test.

Table S4. Uni- and multivariate prognostic analysis of MFS in patients with IBC and patients with nIBC, according to MARCKS/PTEN IHC combination and clinicopathological variables.

Cox, MFS nIBC		*n*	Univariate Odds Ratio (95%CI)	*p*-value	*n*	Multivariate Odds Ratio (95%CI)	*p*-Value
Age, years		355	0.99 (0.98–1.01)	0.492			
Pathological type	lobular vs. ductal	355	1.43 (0.84–2.44)	2.15×10^{-02}	351	1.68 (0.97–2.93)	0.064
	mixed vs. ductal		1.59 (0.73–3.46)		351	1.52 (0.69–3.34)	0.298
	other vs.ductal		0.24 (0.07–0.75)		351	0.35 (0.11–1.12)	0.078
Pathological axillary node status, pN	1 vs. 0	352	3.08 (1.95–4.87)	1.46×10^{-06}	351	2.25 (1.40–3.63)	8.25×10^{-04}
Pathological tumor size (pT)	pT2 vs. pT1	355	2.71 (1.62–4.53)	1.50×10^{-05}	351	1.78 (1.03–3.07)	3.72×10^{-02}
	pT3 vs. pT1		3.83 (2.14–6.85)		351	2.51 (1.38–4.56)	2.59×10^{-03}
Pathological grade	2 vs. 1	354	2.15 (1.24–3.73)	3.42×10^{-04}	351	1.62 (0.91–2.88)	0.100
	3 vs. 1		3.23 (1.82–5.75)		351	2.38 (1.27–4.44)	6.71×10^{-03}
Molecular Subtype	HR+/HER2–vs. HER2+	316	0.75 (0.34–1.64)	0.758			
	TN vs. HER2+		0.83 (0.32–2.13)				
MARCKSneg/PTENpos vs. no-MARCKSneg/PTENpos		231	0.95 (0.52–1.76)	0.882			

Cox, MFS IBC		*n*	Univariate Odds Ratio (95%CI)	*p*-Value	*n*	Multivariate Odds Ratio (95%CI)	*p*-Value
Age, years		115	1.02 (0.99–1.04)	0.173			
Pathological type	lobular vs. ductal	139	1.48 (0.67–3.25)	0.602			
	mixed vs. ductal		0.00 (0.00–Inf)				
	other vs. ductal		0.40 (0.06–2.88)				
TNM, N	1 vs. 0	136	1.86 (1.03–3.36)	4.11×10^{-02}	40	1.48 (0.60–3.66)	0.401
Pathological grade	2 vs. 1	135	0.63 (0.26–1.52)	0.538			
	3 vs. 1		0.80 (0.36–1.79)				
Molecular Subtype	HR+/HER2–vs. HER2+	97	1.04 (0.55–1.95)	0.992			
	TN vs. HER2+		1.04 (0.41–2.58)				
MARCKSneg/PTENpos vs. no-MARCKSneg/PTENpos		43	2.83 (1.26–6.32)	1.14×10^{-02}	40	0.38 (0.16–0.90)	2.81×10^{-02}

*, the *p*-value is for the Wald test.
